# Laser interventions in coloproctology. A plea for standardized treatment protocols

**DOI:** 10.1007/s10151-023-02859-2

**Published:** 2023-08-28

**Authors:** P. C. Ambe

**Affiliations:** 1 Department of General, Visceral Surgery and Coloproctology, Vinzenz-Pallotti-Hospital Bensberg, Bergisch Gladbach, Germany; 2 Department of Health, Faculty of Medicine, Witten/Herdecke University, Witten, Germany

## Background

Benign proctologic conditions including hemorrhoids, fistula in ano, anal fissures and pilonidal sinus constitute a significant portion of the workload in general and colorectal surgery. Over the last decade, a laser-associated management option for these entities has been largely thematized, and many surgeons have added laser-based techniques to their treatment armamentarium [1, 2]. The most commonly used laser systems in the literature include the CO_2_ [3], neodymium YAG [4] and diode lasers [5]. In recent years, increasingly more papers have been published on the use of diode laser in the management of proctologic disorders. The issues raised in this manuscript are based on the experience with the Leonardo® diode laser, Biolitec Biomedical Technology GmbH, Jena, Germany.

## Laser hemorrhoidoplasty (LHP)

High-quality data are currently available for the use of the diode laser in the management of hemorrhoids, a technique commonly known as laser hemorrhoidoplasty (LHP) [6–8]. To perform LHP, the laser fiber is inserted via a 2-mm incision at the anocutaneous line and gently advanced in the submucous layer of the anorectum. Using visual control via an indicator light as well as digital control via a palpating finger, the laser fiber is advanced to 2–3 cm above the dentate line. At this point, laser energy is placed at three adjacent points. Hereafter, the fiber is gently withdrawn to the level of the dentate line, where three impulses are given. The procedure is completed by 2–3 impulses below the dentate line. The other piles are treated in a similar manner. The energy leads to shrinkage of the pills with subsequent fixation onto the submucosa. LHP does not including resection and is therefore an organ- and function-preserving procedure.

## Fistula-tract laser closure (FiLaC)

The fistula-tract laser closure (FiLaC procedure) is increasingly being employed for the management of both cryptoglandular fistula in ano and fistula in Crohn's [9–11]. The laser fiber is brought into the fistula tract and is steadily slowly withdrawn while applying the laser energy directly on the epithelized fistula tract in a 360° (ringlike) radiation manner. While the closure of the internal opening is still a matter of debate among experienced users, widening the external opening is uniformly performed by all users to enable optimal drainage. The need of curettage or irrigation of the fistula tract prior to performing FiLaC is also a matter of debate. There is an increasing number of data, mostly from single-center retrospective studies looking especially at the healing rate and risk of postoperative continence disturbance following FiLaC [12–14]. Current rates of healing of about 65–70% have been reported in recent systematic reviews following one FiLaC attempt in cryptoglandular fistula [15, 16]. This increases to about 80% after a redo-FiLaC. A very encouraging healing rate of about 55% was recently reported for Crohn's fistula in a systematic review by Cao et al. [17].

## Sinus laser associated closure (SiLaC)

This is a minimally invasive procedure including pit picking to remove the hairs from the sinus with or without curettage and irrigation, followed by closure of the communicating sinus tracts using laser energy similar to the FiLaC procedure [18, 19]. Published data from single institutional retrospective collectives uniformly indicate high success rates ranging from 72–96% from just one treatment [18–20]. SiLaC can be readily repeated in case recurrence or treatment failure.

## Laser fissuroplasty (LaFiP)

This procedure uses the laser to manage chronic anal fissures. Currently, there is just a handful of publications on the use of laser for this indication [21]. Success rates in the limited publications are very encouraging [21, 22].

## Many unanswered questions

In the recently held Second Proctocom meeting organized by Biolitec in Malaga, Spain, from June 9 to 10 2023, many significant issues were raised regarding laser application in proctology. In light of the constantly growing number of surgeons applying laser procedures in clinical practice as well as a constantly increasing number of scientific publications in this field, relevant controversies regarding pre-, peri- and postoperative management standards were extensively discussed.

The optimal energy for each indication appears to be a major perioperative concern. Although Biolitec Biomedical Technology GmbH has clear recommendations for the most common indications (LHP, FiLaC and SiLaC) for its laser products, a huge heterogeneity still exists among users in daily practice. Though not primarily linked to the laser procedure per se, the role of preoperative bowel prepping in patients undergoing laser proctologic procedures is still to be defined. Similarly, the needs for perioperative antibiotics beyond “single shot” and postoperative antithrombosis prophylaxis also represent unaddressed issues in this particular treatment domain. In addition, there is a high degree of variation in follow-up (when, how and for how long) and in the definition of healing for almost all laser-based procedures in coloproctology.

Although the above-mentioned questions are prima vista relevant for all proctologic indications managed with the Biolitec diode laser, other procedure-specific issues need to be addressed. In LHP, for example, it is unclear whether all piles should be managed at the same time. Also, the indication to manage skin tags and how to go about this are not clear. Some surgeons do use other procedures like hemorrhoidal desarteralization, hemorroidal artery ligation (HAL), mucopexy or rectoanal repair (RAR) in combination with LHP. While these additional procedures may improve the immediate postoperative results, it becomes difficult to objectively define the role of LHP in the overall treatment success.

Regarding FiLaC, specific issues including the role of a draining seton and the need of imaging prior to definite fistula closure (FiLaC) constitute important issues to address. Also, intraoperative conditioning of the fistula tract, e.g., via irrigation (with saline or peroxide) and/or curettage prior to laser application, needs some clarification. Closing the internal orifice or not, and, if yes, how to do that (simple Z-stich vs. flap vs. clip), represents another relevant determinant for success that needs to be addressed.

In SiLaC curettage and jet irrigation with the aim of removing entrapped hairs and destroying epithelizing sinus walls may follow pit picking. While the rationale behind these additive measures sounds plausible, curettage in particular may increase the size of the sinus and thus reduce the efficacy of treatment by impairing the effective transfer of the laser energy onto the walls of the sinus/tract. Also, a high degree of heterogeneity exists regarding the number of pits that need to be opened or excised.

Looking at LaFiP, the need to excise the ulcer prior to performing laser is a topic of controversy. Also the management of the hypertrophic anal papilla and the sentinel pile remains a topic of discussion. The need of additive measures, e.g., botox injection, application of topical remedies (e.g., diltiazem) and lateral sphincterotomy, and how to define success represent hot topics that need to be addressed.

## Summary

The advantages of laser-based procedures in coloproctology are readily identifiable. The minimally invasive nature of these procedures with organ/tissue preservation is associated with less pain and high patient comfort. Small incisions enable quick recovery and an early return to work [23, 24]. Also the tissue-preserving nature of these procedures enables good postoperative functionality especially regarding continence. In addition, laser-based procedures in coloproctology are easy to learn.

Despite the above-mentioned advantages of laser procedures, there is a wide variation in performing standard procedures among users. While these variations are definitely encouraged in the hands of experienced users, there is need to standardize treating protocols for surgeons at the beginning of their learning curves. In addition, standardizing treatment protocols would enable a better comparison of results from different institutions. This is especially true regarding the need to generate more data to study the efficacy of these new techniques in the management of common conditions.

## Conclusion

Standardized treatment protocols for the most common indications (LHP, FiLaC and SiLaC) are needed. Therefore, a guideline development group of international surgeons with experience in laser-based procedures in coloproctology should be initiated with the aim of creating treatment protocols.

## Data Availability

Not applicable.

## References

[1] Gupta K (2022). Lasers in surgery: from past to present. Lasers in Proctology.

[2] Rahman AT (2019). Laser in proctology a new hope in treating the distressing anal diseases. Faridpur Med Coll J.

[3] Sowula A (2010). The role of laser CO2 in proctology. Wiadomosci Lekarskie (Warsaw, Poland: 1960).

[4] Marcon N, Haber G, Kortan P (1986) Neodymium YAG Laser for the Treatment of Anal Condylomata. In Laser/optoelectronics in medicine/laser/optoelektronik in der medizin: Proceedings of the 7th International congress/Vorträge des 7. Internationalen kongresses laser 85 optoelektronik mit/with 2nd international Nd: YAG laser conference. Springer.

[5] Bachtsetzis G (2019). ELITE: a diode laser minimal invasive technique for hemorrhoids during the surgical treatment for anal fissure. J Surg Case Reports.

[6] Weyand G (2017). Laserhemorrhoidoplasty with 1470 nm diode laser in the treatment of second to fourth degree hemorrhoidal disease-a cohort study with 497 patients. Zentralbl Chir.

[7] Poskus T (2020). Results of the double-blind randomized controlled trial comparing laser hemorrhoidoplasty with sutured mucopexy and excisional hemorrhoidectomy. Int J Colorectal Dis.

[8] Wang D (2005). Effect of diode laser coagulation treatment on grade III internal hemorrhoids. Zhonghua wei Chang wai ke za zhi Chin J Gastrointest Surg.

[9] Alam A (2020). FiLaC® and Crohn’s disease perianal fistulas: a pilot study of 20 consecutive patients. Tech Coloproctol.

[10] Giamundo P (2014). Closure of fistula-in-ano with laser–FiLaC™: an effective novel sphincter-saving procedure for complex disease. Colorectal Dis.

[11] Wilhelm A, Fiebig A, Krawczak M (2017). Five years of experience with the FiLaC™ laser for fistula-in-ano management: long-term follow-up from a single institution. Tech Coloproctol.

[12] Serin KR (2020). Retrospective analysis of primary suturing of the internal orifice of perianal fistula during FiLaC procedure. Surg Laparosc Endosc Percutan Tech.

[13] Wolicki A (2021). Sphincter-saving therapy for fistula-in-ano: long-term follow-up after FiLaC®. Tech Coloproctol.

[14] Yöntem AFTSK, Kapatılması LYFT (2017). Closure of fistula tract with FiLaC™ laser as a Sphincter-Preserving method in anal fistula treatment. Turk J Colorectal Dis..

[15] Frountzas M (2020). Could FiLaC™ be effective in the treatment of anal fistulas? A systematic review of observational studies and proportional meta-analysis. Colorectal Dis.

[16] Elfeki H (2020). A systematic review and meta-analysis of the safety and efficacy of fistula laser closure. Tech Coloproctol.

[17] Cao D (2022). Efficacy and safety of FiLaC™ for perianal fistulizing Crohn’s disease: a systematic review and meta-analysis. Tech Coloproctol.

[18] Pappas A, Christodoulou D (2018). A new minimally invasive treatment of pilonidal sinus disease with the use of a diode laser: a prospective large series of patients. Colorectal Dis.

[19] Dessily M (2017). Pilonidal sinus destruction with a radial laser probe: technique and first Belgian experience. Acta Chir Belg.

[20] Ganduboina R (2023). Laser ablation: a unique and beneficial therapeutic option for pilonidal sinus? And the potential for further innovation—a review. Lasers Med Sci.

[21] Esfahani MN, Madani G, Madhkhan S (2015). A novel method of anal fissure laser surgery: a pilot study. Lasers Med Sci.

[22] Fateh SH (2016). Outcome of laser therapy in patients with anal fissure. Hamdan Med J.

[23] Wee IJY (2023). Laser hemorrhoidoplasty versus conventional hemorrhoidectomy for grade II/III hemorrhoids: a systematic review and meta-analysis. Ann Coloproctol.

[24] Lie H (2022). Laser hemorrhoidoplasty for hemorrhoidal disease: a systematic review and meta-analysis. Lasers Med Sci.

